# COVID-19 Related Myocarditis and Myositis in a Patient with Undiagnosed Antisynthetase Syndrome

**DOI:** 10.3390/biomedicines11010095

**Published:** 2022-12-30

**Authors:** Daniel Duda-Seiman, Nilima Rajpal Kundnani, Daniela Dugaci, Dana Emilia Man, Dana Velimirovici, Simona Ruxanda Dragan

**Affiliations:** 1 Department of Cardiology, “Victor Babes” University of Medicine and Pharmacy, 300041 Timisoara, Romania; 2 Department of Functional Sciences, Physiology, Center of Immuno-Physiology and Biotechnologies (CIFBIOTEH), “Victor Babes” University of Medicine & Pharmacy, 300041 Timisoara, Romania; 3 Institute of Cardiovascular Diseases, 300310 Timisoara, Romania

**Keywords:** myocarditis, COVID-19, myositis, autoimmune diseases

## Abstract

Background: The clinical presentation of SARS-CoV-2 varies from patient to patient. The most common findings noted were respiratory tract infections, of different severity grades. In some cases, multi-organ damage was noted. Due to its high potential for causing severe systemic inflammation such as myositis and myocarditis, patients should be properly investigated, which carries high chances of SARS-CoV-2 being easily missed if not investigated on time and which can result in more fatal outcomes. Case report: We present a case of COVID-19 infection in a non-vaccinated male patient, who presented to our clinic with no symptoms of respiratory involvement but with severe muscle aches. Cardiac markers and procalcitonin levels were high, and concentric hypertrophy of the left ventricle, severe hypokinesia of the interventricular septum and of the antero-lateral wall, hypokinesia of the inferior and posterior wall and an ejection fraction of the left ventricle being around 34% was noted. Coronary angiography showed no lesions. Corticosteroids and antibiotics were instituted which showed improvement. A possible link to an autoimmune process was suspected, due to the presence of anti-PL-7 antibody, suggesting an antisynthetase syndrome. Conclusion: Each and every patient should be thoroughly investigated, and presently little is known in regards to this virus. Studies focusing on possible relationships between the COVID-19 and autoimmune disease can help to potentially generate better outcomes.

## 1. Introduction

COVID-19 was declared a global pandemic after the first case was detected in 2019, which led to a massive spread worldwide, infecting and affecting lives of millions of people with very high mortality rates [1,2,3]. Healthcare system around the globe were not prepared to fight this unprecedented situation, especially because very little was known about it, and no vaccinations or medications to prevent it were on hand. Many changes were instituted to separate entry and exit of the infected cases in most hospitals. WHO guidelines were issued to stop the spread of this devastating virus [3,4]. Surgical interventions were done only on an emergency basis, and elective surgeries were postponed [5]. The clinical presentation varied widely depending on different strains of the virus [6,7]. Few patients were found to be asymptomatic, while some had serious respiratory symptoms, to the extent that oxygen therapy was mandatory, while some developed multi-organ failure [7]. The vaccination programs were implemented as soon as they were approved by the WHO to minimize the spread. The use of pharmacological and non-pharmacological measures were evaluated and implemented on a large scale to combat the pandemic [8].

Angiotensin-converting enzyme 2 (ACE2) is crucial in cardiovascular neurohumoral regulation. The increased binding affinity of the SARS-CoV-2 virus to ACE2 receptors modifies the ACE2 signaling pathways, leading to acute myocardial injuries [9]. The cytokine release syndrome is the prerogative of severe forms of COVID-19. This extreme inflammatory response with high levels of cytokines determines systemic injuries, including endothelial and myocardial ones, as well as acute respiratory distress syndrome and various end-organ damage. Viral myocarditis might appear as a result of a direct myocardium infection [10]. From 8–62% of COVID-19 hospitalized patients show increased levels of cardiac troponins, as a consequence of acute cardiac injury. If echocardiographic changes are present, there is an increased risk of in-hospital mortality, which, fortunately, did not happen in our case. Common echocardiographic abnormalities are: left ventricular wall motion changes, global left ventricular systolic dysfunction, right ventricular dysfunction, pericardial effusion, and diastolic dysfunction [11]. There is a large clinical variety of presentations of COVID-19 myocarditis: from mild symptoms (fatigue, dyspnea), to severe situations with hemodynamic instability. Recent literature illustrates the possibility of variable evolution of the subject with myocardial injury in the context of SARS-CoV-2 infection. Patients with no or mild cardiac symptoms at the onset might develop acute heart failure and cardiogenic shock [12]. The Antisynthetase Syndrome (ASS) is a rare condition belonging to the Idiopathic Inflammatory Myopathies (IMS), which is quite difficult to diagnose. Its clinical presentation is heterogenous, with interstitial lung disease and/or inflammatory myositis, and with positive antisynthetase antibodies [13].

Here we present a non-vaccinated, infected COVID-19 male, who developed serious cardiac complications. 

## 2. Case Details

**Figure 1:**
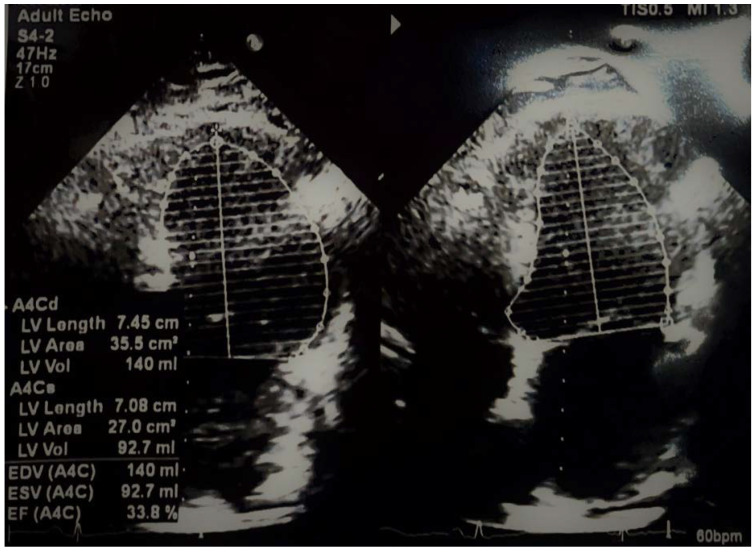
Apical 4-chambers (A4C) echocardiography: moderately decreased left ventricular ejection fraction (LV, left ventricle; EDV, end-diastolic volume; ESV, end-systolic volume; EF, ejection fraction).

**Figure 2:**
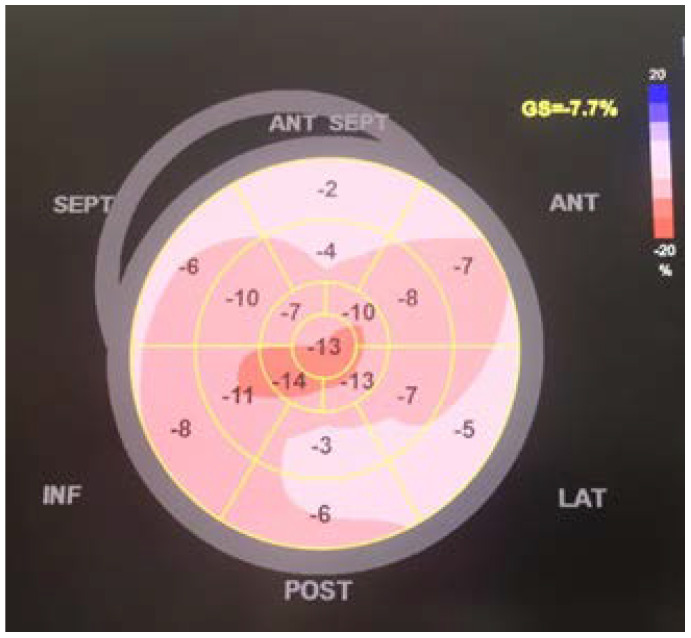
Global strain (GS) of the left ventricle: polar map with the regional values and the GS value calculated from the 17 segments of the left ventricle, which is significantly impaired (ANT, anterior; ANT SEPT, anteroseptal; GS, global strain; INF, inferior; LAT, lateral; POST, posterior; SEPT, septal).

**Figure 3:**
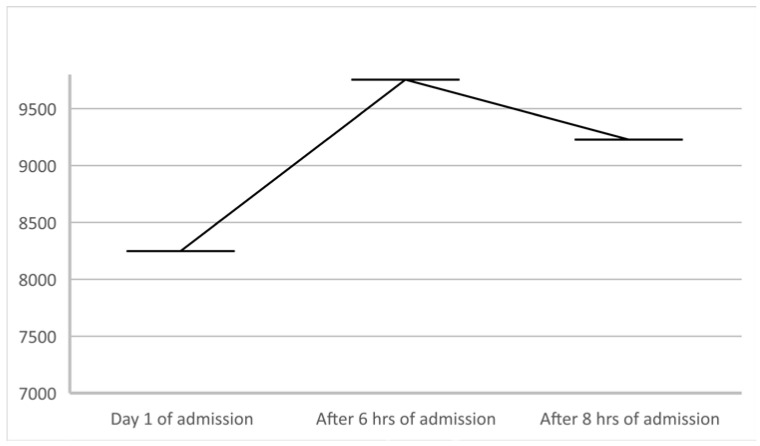
hsTnI (high sensitive I troponin) behavior during hospitalization.

**Figure 4:**
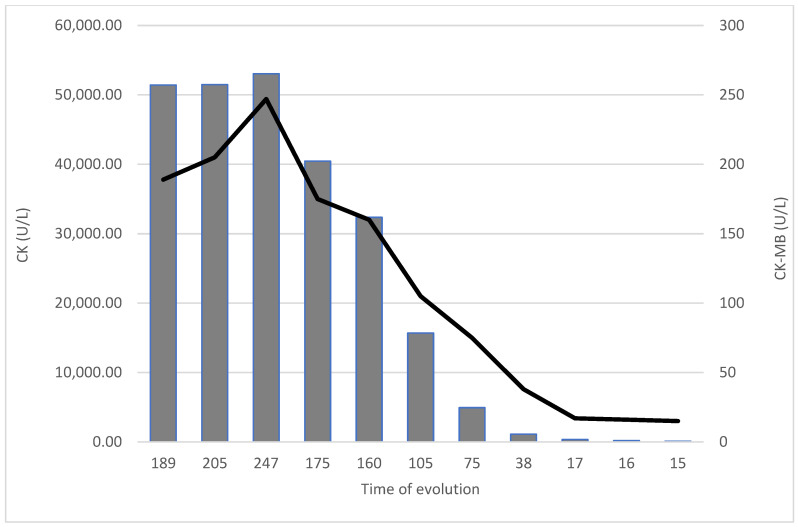
CK and CK-MB behavior during hospitalization. (CK, creatine phosphokinase; CK-MB, muscle-brain creatine phosphokinase isoenzyme).

A 62-year-old male patient, known to have primary hypertension and type 2 diabetes mellitus, presented with a brutal onset of loss of consciousness, muscle pain in the upper limbs accompanied by increased movement impairment. In the emergency department a rapid antigen test for SARS-CoV-2 was performed which was found to be positive. The patient had not been vaccinated against SARS-CoV-2. Clinical assessment showed no significant changes: BMI = 32.41 kg/m^2^; BP = 140/85 mmHg; HR = 95 bpm with normal rhythmic heart beats; SpO_2_ = 97% (room air); no fever; no pulmonary rales. Initial lab values showed increased inflammation (CRP = 59.5 mg/L), increased values of cardiac enzymes (hsTnI = 8248 ng/L) and possible sepsis (procalcitonin = 68.03 ng/L). The ECG showed no acute ischemic changes. A CT angiography of the pulmonary arteries was performed with the following result: cardiomegaly with contrast refluxed into the hepatic veins, pulmonary arteries with dimensions at the upper limits for normal values, homogeneously opacified, without acute pulmonary lesions. Considering these data, the patient underwent a standard cardiac ultrasound examination revealing concentric hypertrophy of the left ventricle, severe hypokinesia of the interventricular septum, of the anterior and antero-lateral wall, and hypokinesia of the inferior and posterior wall with an estimated ejection fraction of the left ventricle to be approximately 34% (Figure 1); global strain was −7.7% (Figure 2); systolic pressure in the pulmonary artery was 50 mmHg. Approximately 2 h after admission, the dynamics of myocardial necrosis was entertained, enzymes registered an increasing trend (hsTnI = 9755 ng/mL; CK = 51432 U/L; CK-MB = 189 U/L). As a result of the accumulated data, an acute coronary syndrome without ST segment elevation was suspected. The patient underwent coronary angiography using the right radial artery approach, in which the coronary arteries revealed no significant angiographic lesions. Figure 3 and Figure 4 show the dynamics of hsTnI, CK and CK-MB.

**Figure 5:**
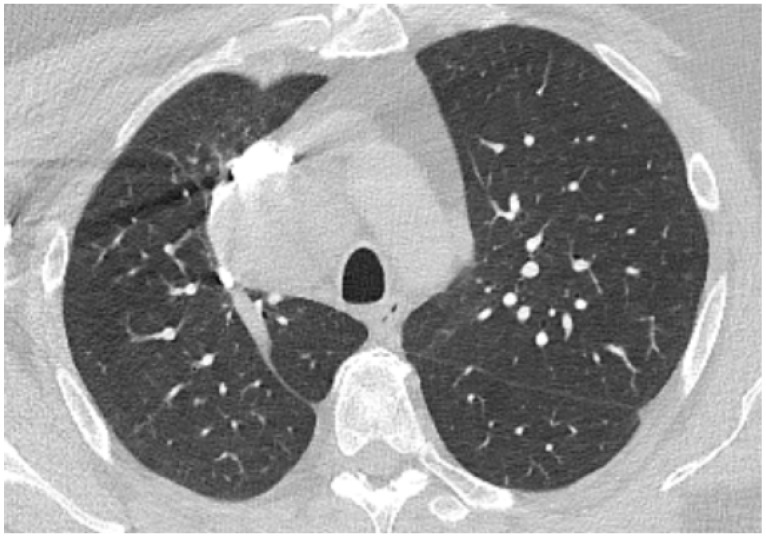
Native chest computer-tomography in the context of confirmed SARS-CoV-2 infection: fine areas of ground glass arranged peripherally classified as minimal lung damage.

Considering possible sepsis of unknown origin, antibiotics were initiated with ceftriaxone. A sputum culture was positive for Klebsiella pneumoniae spp pneumoniae, and ceftriaxone was continued according to the antibiogram. Blood cultures taken consecutively were negative. During day 3, a thorax CT scan was performed, which showed fine areas of ground glass arranged peripherally and classified as minimal lung damage (Figure 5) and small bilateral areas of pleurisy with a maximum thickness of 10 mm in the right costo-phrenic recess. Methylprednisolone was given, with progressive decreasing of the dose over time. Standard medication for heart failure with a reduced ejection fraction was given [14]. The serum level of interleukin-6 was 2.32 pg/mL, which was considered to be normal [15]. The evolution was favorable: cardiac enzymes, inflammatory markers and procalcitonin continued to decrease and eventually were normalized. Kidney function was preserved. The muscle pain in the upper limbs subsided, with full recovery of functionality. Patient tested negative for SARS-CoV-2 infection on day 14 (RT-PCR).

Corroborating the clinical, paraclinical and biological context, the panel of IgG antibodies specific for myositis was observed, with a positive result for anti-PL-7 antibodies. We consider that the episode of myocarditis and extensive myositis was clinically triggered by the SARS-CoV-2 infection, possibly linked to his autoimmune status, which was unknown to the patient. 

## 3. Discussion

This is a case of a patient diagnosed with COVID-19, not vaccinated against SARS-CoV-2 infection, with no respiratory symptoms, but with the presence of an inflammatory syndrome with skeletal and myocardial muscle damage. Intriguingly, symptomatology due to myocarditis and heart failure was lacking and a bacterial pulmonary superinfection did not produce classic clinical symptoms such as dyspnea, cough or fever. Laboratory assessment documented sepsis. Cytokine storm was not documented and specific biologic therapy was not considered. 

As previously described [16], COVID-19 may be associated with an extreme inflammatory response of both skeletal muscles and the myocardium, with respiratory symptoms being poorly expressed or even absent. We considered that the muscle damage in this case was due to SARS-CoV-2 infection, previously reported by Pawar et al., who observed similar findings in their case study [17]. Myositis and myocarditis were reported, but post vaccination, which in our case, could not be correlated as our patient was unvaccinated [18]. Given its severity, we also considered investigating the potential of an underlying autoimmune condition for myositis for which the anti-PL-7 antibodies were positive. Of course, a muscular biopsy would have been necessary to further document the findings, but the patient refused. Myalgia and muscular weakness can be found quite frequently in COVID-19 patients (11–50%) [19], but an association with a connective tissue disorder is rare. It has been found that patients positive for anti-PL-7 or anti-PL-12 antibodies may develop a severe form of interstitial lung disease with myositis not being a frequent association [13]. More evidence is evolving, illustrating the fact that SARS-CoV-2 may induce different myopathies, while anti-PL-7 positive patients may clinically express dermatomyositis, during the course of a COVID-19 infection [20]. In the case of patients with known and therapy-controlled ASS, healing for COVID-19 infection was achieved if they were asymptomatic for acute COVID-19 and had at least one negative SARS-CoV-2 polymerase chain reaction test. Long-term evolution after COVID-19 in unvaccinated patients with ASS may be characterized by a worsening of the underlying condition (pulmonary hypertension, myocarditis) [21]. Momin and Nagori [22] presented the case of a female patient with family exposure to COVID-19 infection and vaccinated with the second dose of a mRNA vaccine. She developed symptoms (body ache/pain, dyspnea and leg swelling), but testing for SARS-CoV-2 infection was negative. Thorax computer tomography was suggestive for COVID-19. Laboratory findings were consistent for ASS. As in our case, ASS might be revealed by exposure to SARS-CoV-2 infection, corticosteroids being the treatment of choice for clinical and biological improvement.

Myositis-specific autoantibodies and myositis-associated autoantibodies should be determined in COVID-19 patients who present with clinical myositis. Autoimmunity might be caused by molecular triggers, or by a potential mechanism which primes an underlying predisposition, or by the COVID-19 induced immune dysfunction [23].

Cortico-therapy is beneficial by decreasing rhabdomyolysis and the inflammatory syndrome, consistent with literature data [13,16,19,20]. The COVID-19 pandemic led to increased diagnosis of anti-synthetase syndromes [24], which needs more investigation into the link between SARS-CoV-2 infections and autoimmune diseases.

## 4. Conclusions

Since the nature and clinical pattern is unclear and differs from patient to patient, physicians should leave no stone unturned while dealing with a COVID-19 positive patient. A thorough investigation is a must even in the absence of classical symptoms. More extensive studies are still required to better understand the link between the virus and different pathologies, especially in patients suffering from autoimmune diseases.

## Data Availability

Data will be provided upon written request.
